# Supracondylar rotation osteotomy of the femur influences the coronal alignment of the ankle

**DOI:** 10.1186/s40634-021-00340-3

**Published:** 2021-04-20

**Authors:** Christian Konrads, Marc-Daniel Ahrend, Myriam R. Beyer, Ulrich Stöckle, Sufian S. Ahmad

**Affiliations:** 1 Department for Trauma and Reconstructive Surgery, BG Klinik, University of Tübingen, Tübingen, Germany; 2 Center for Musculoskeletal Surgery, Charité - University Medical Center Berlin, Berlin, Germany

**Keywords:** Derotation, Torsional alignment, Long leg axis, Anterior knee pain, Realignment

## Abstract

**Purpose:**

Osteotomies represent well-established treatment-options for the redistribution of loads and forces within and around the knee-joint. Effects of these osteotomies on the remaining planes and adjacent joints are not fully understood. The aim of this study was to determine the influence of a distal-femoral-rotation-osteotomy on the coronal alignment of the ankle. It was hypothesized that supracondylar-external-rotation-osteotomy of the distal femur leads to a change in the coronal orientation of the ankle joint.

**Methods:**

Long-leg standing radiographs and CT-based torsional measurements of 27 patients undergoing supracondylar-rotational-osteotomy of the femur between 2012 and 2019 were obtained and utilized for the purpose of this study. Postoperative radiographs were obtained after union at the osteotomy-site. The hip-knee-ankle-angle (HKA), the mechanical-lateral-distal-femur-angle (mLDFA), and Tibia-Plafond-Horizontal-Orientation-Angle (TPHA) around the ankle were measured. Comparison between means was performed using the Wilcoxon-Mann–Whitney test.

**Results:**

Twenty-seven patients with high femoral antetorsion (31.3° ± 4.0°) underwent supracondylar-external-rotation-osteotomy. The osteotomy led to a reduced antetorsion (17.4 ± 5.1; *p* < 0.001) and to a valgisation of the overall limb-alignment. The HKA decreased by 2.4° ± 1.4° (*p* < 0.001). The TPHA decreased by 2.6° (*p* < 0.001).

**Conclusions:**

Supracondylar external rotation osteotomy of the femur leads to lateralization of the weight bearing line at the knee and ankle due to valgisation of the coronal limb alignment. The mobile subtalar joint has to compensate (inversion) for the resulting valgus orientation of the ankle to ensure contact between the foot and the floor. When planning a rotational osteotomy of the lower limb, this should be appreciated – especially in patients with a preexisting valgus alignment of the lower extremities or restricted mobility in the subtalar joint.

## Background

Osteotomies around the knee represent powerful modalities for the treatment of bony deformities and degenerative joint disease. The intended effects of these osteotomies act on joints by redistributing loads and force vectors. Rotational osteotomies of the femur influence the overall orientation of the femoral antetorsion and demonstrate an influence on all major joints of the lower extremities: hip, knee, and ankle. The vectors of the quadriceps muscle are ultimately altered by a rotational osteotomy of the femur, subsequently influencing lateral force vectors acting on the patella. Furthermore, the orientation of the femoral neck in space is also influenced by femoral anteversion. Clear evidence linking torsional abnormalities of the femur to hip and patellofemoral complains is present [1–4].

It has been demonstrated earlier that a supracondylar external rotational osteotomy of the femur leads to a valgisation, which can be measured on standing long-leg radiographs using the mechanical lateral distal femoral angle (mLDFA) [5]. It is therefore important to consider all possible effects of an osteotomy during surgical planning and expand planning beyond the main plane of interest. This would reduce the likelihood of creating an unwanted conflict on a different level. The effects of femoral supracondylar rotation osteotomies on the ankle have not been studied before.

This study deals with the influence of a supracondylar rotation osteotomy of the distal femur on the coronal limb alignment with special focus on the ankle. As the orientation of the femoral curvature is likely to change during a rotation osteotomy, we ask the question of whether this might alter the coronal ankle alignment.

The aim of this study was to retrospectively determine the influence of supracondylar rotation osteotomies of the femur on the long-leg axis in the frontal plane around the knee and the ankle. We hypothesized that supracondylar external rotation osteotomy of the femur leads to significant valgisation of the knee and ankle joints.

## Materials and methods

This retrospective study was approved by our university’s Ethics Committee.

### Patients

**Fig. 1:**
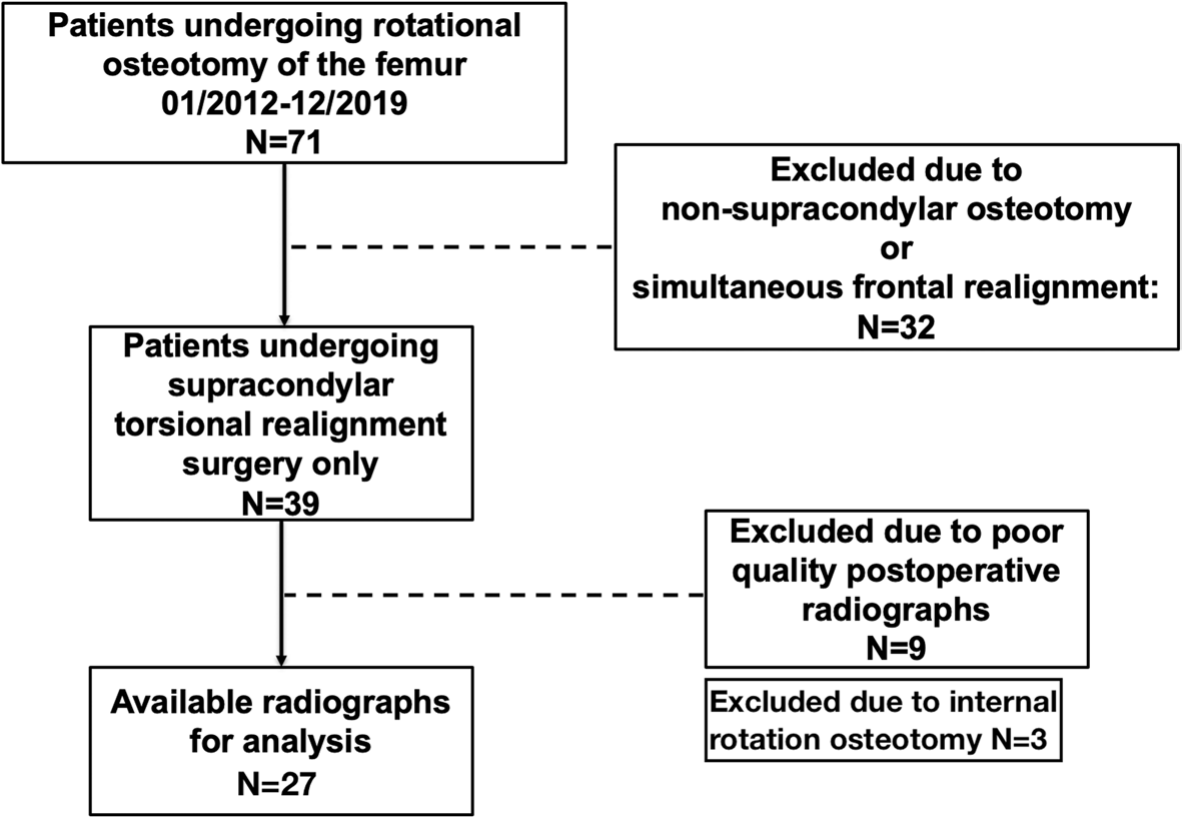
Flowchart demonstrating inclusion

Patients undergoing supracondylar external rotation osteotomy of the femur were considered eligible for inclusion in the study, provided that sufficient pre- and postoperative radiographs were available. Exclusion criteria were: correction in a plane other than the axial plane, long-leg standing radiograph not available, postoperative fracture of a lower leg or acetabulum prior to osteotomy consolidation, or instability of the knee or ankle due to postoperative trauma. Further, exclusion was necessary, if X-ray quality was defined as inferior, or in the case of missing consent regarding the utility of clinical data. Considering the above criteria, 27 legs of 26 patients undergoing osteotomy were considered eligible for inclusion in the study (Fig. 1).

### Surgical procedure

All osteotomies were planned using a landmark based deformity analysis [6, 7]. In axial CT images, the orientation of the femoral neck was measured against a horizontal line on the computer screen. Then the posterior condylar line at the distal femur was measured against the horizontal line. The two angles had to be either added (when oriented in different directions) or subtracted (when oriented in the same direction) to generate a value for femoral torsion.

Surgeries were performed by three experienced surgeons in a standardized manner. The patient was positioned supine. Two Schanz screws were positioned, one proximal and one distal to the osteotomy level. They were inserted in angulation to each other in the amount of the planned correction angle. A medial subvastus approach was established as described earlier [3, 8]. Supracondylar monoplane osteotomy was performed and a TomoFix MDF plate (DePuy Synthes, Solothurn, Switzerland) was used for fixation [9].

### Radiographs

With the aim to ensure a standardized radiography, long-leg weight-bearing radiographs were obtained in accordance to Paley with a 1.3 m cassette (Global Imaging Baltimore, MD) [10]. Long leg antero-posterior standing radiographs were obtained with the patient standing in a bipedal stance in front of the long film cassette. Radiologic technical assistants were instructed to position both legs with the patella centered between the femoral condyles. The radiography tube was positioned in a distance of 305 cm. The selected film cassette was of sufficient length to include the hips, knees, and ankles. The magnification with this setup was approximately 5%. A calibration device (250 mm steel ball) was used to calibrate the radiographs. The X-ray beam was centered on the level of the knee joints.

Femoral torsion was measured using axial CT slides. As multiple methods for measuring femoral torsion exist [7, 11], instead of using the simpler method by Jarrett [6], we measured the femoral torsion according to Waidelich [12] because of its high reliability and especially because norm values are available for this method [12, 13]. For patients older than 18 years, femoral internal torsion of 20° ± 9° and tibial external torsion of 33° ± 8° is considered as normal. The intra-individual difference of both legs is considered as normal up to 4° ± 2° in the femur and 6° + 4° in the tibia [12]. The intra- and interrater reliability using the method of Waidelich is very good: about 2.4° with an ICC of 0.90.

**Fig. 2:**
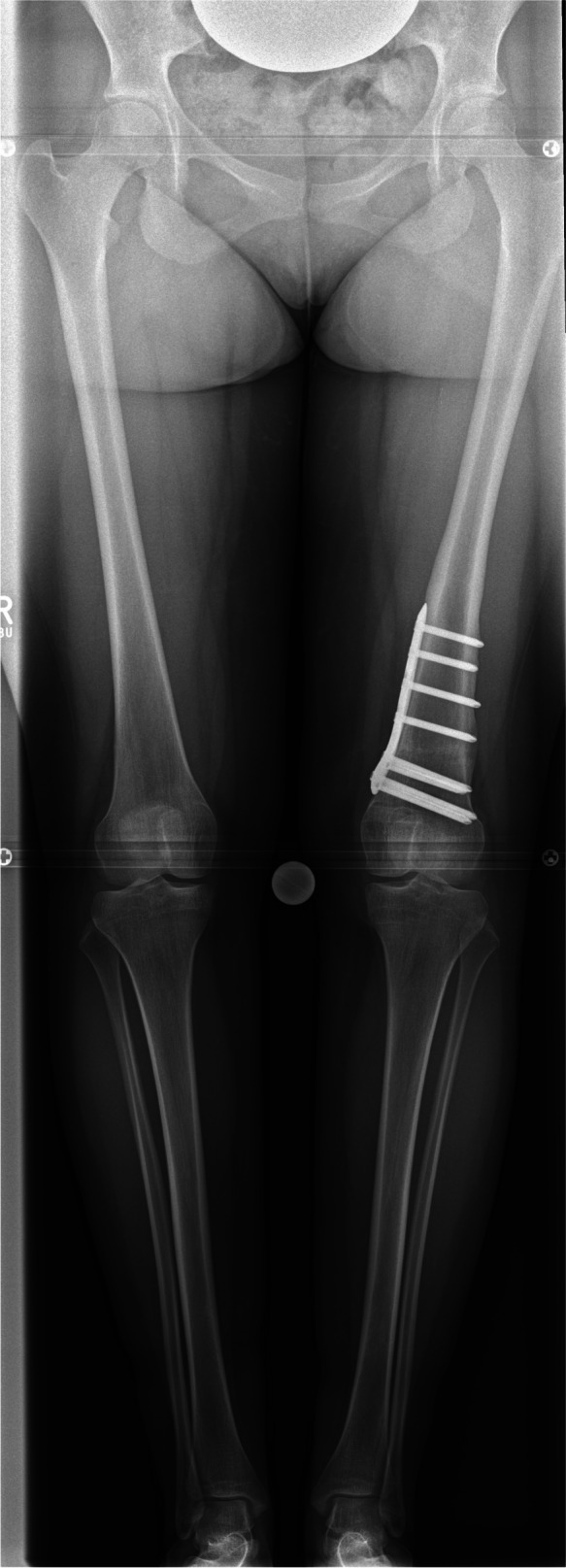
Antero-posterior long-leg weight-bearing radiograph after supracondylar derotation (= external rotation) osteotomy of the femur

All radiographic images were obtained prior to surgery for planning of the deformity correction and were repeated postoperatively after union at the osteotomy site and recovery of limp-free full weight-bearing (Fig. 2).

### Radiographic parameters

**Fig. 3:**
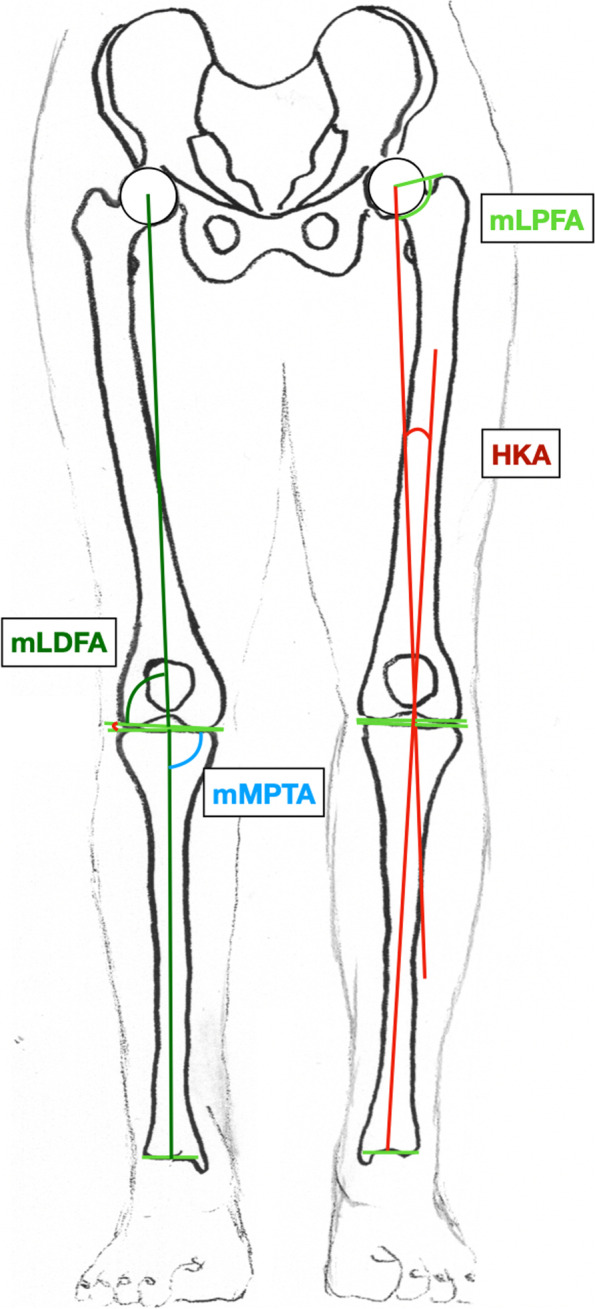
Illustration of the radiographic parameters measured on a long-leg standing X-ray with the knees pointing forward. Measures around the hip and the knee. HKA: Hip Knee Ankle angle, mLDFA: Mechanical lateral distal femoral angle, mLPFA: Mechanical lateral proximal femoral angle, mMPTA: Mechanical medial proximal tibial angle

Radiographic parameters were determined with an accuracy of 0.1 mm using mediCAD® (Hectec, Landshut, Germany). The following parameters were assessed in accordance to Paley [6]:

- Mechanical medial proximal tibial angle (mMPTA)
- Mechanical lateral distal femoral angle (mLDFA)
- Mechanical lateral proximal femoral angle (mLPFA)
- Anatomic Mechanical Angle of the femur (AMA)
- Femoral torsion
- Hip Knee Ankle (HKA) angle, refers to the angle between mechanic axes of the femur and the tibia (Fig. 3). A synonym for HKA is the mechanical tibiofemoral angle (mTFA).

**Fig. 4:**
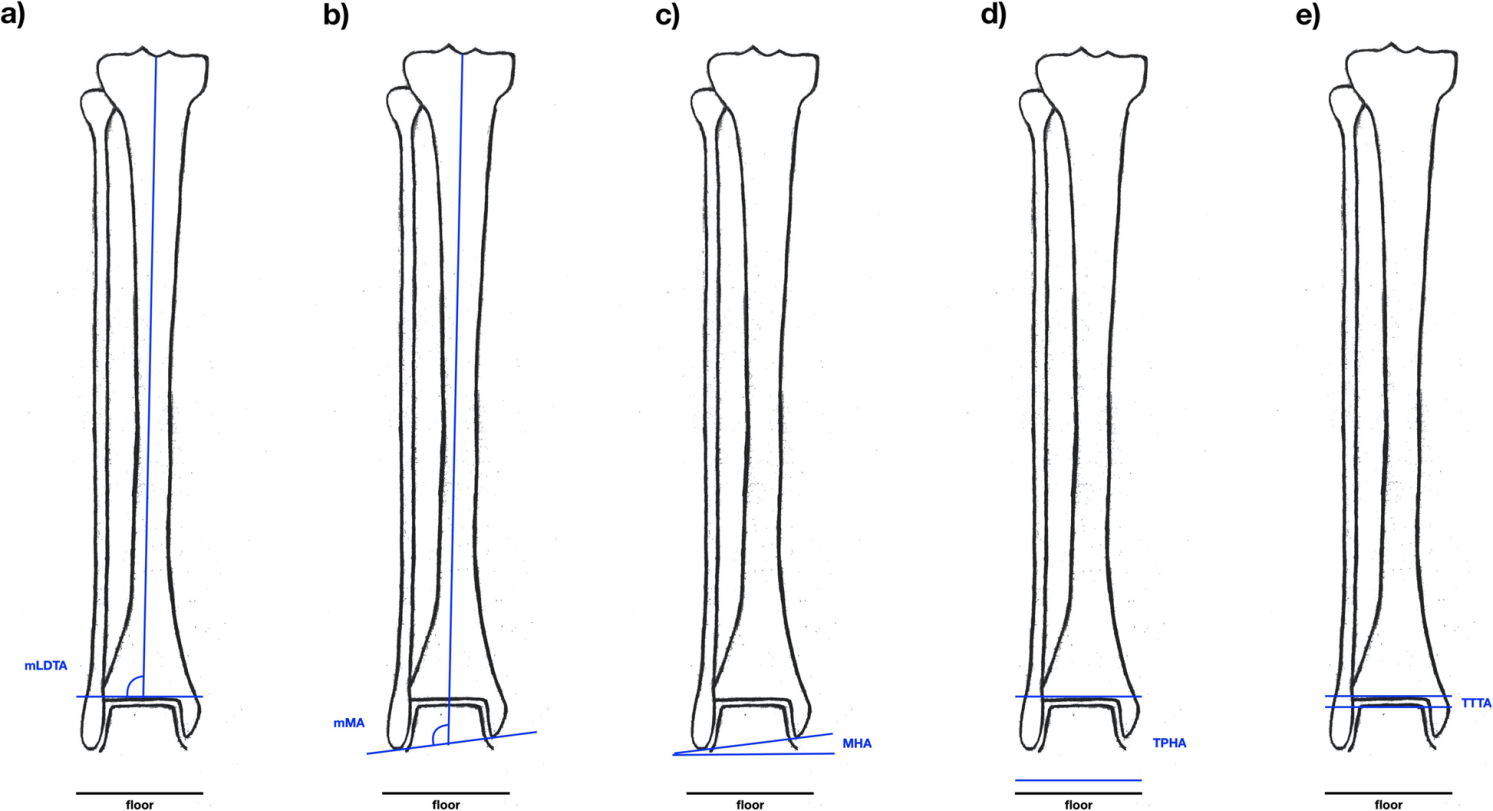
Illustration of the radiographic parameters measured on a long-leg standing X-ray with the knees pointing forward. Measures around the ankle. **a** mLDTA: angle between tibiaplafond and mechanical tibia axis. **b** mMA: angle between malleolar tips and mechanical tibia axis. **c** MHA: angle between malleolar tips and floor. **d** TPHA: angle between tibiaplafond and floor. **e** TTTA: angle between tibio-talar joint surfaces*. *mLDTA: Mechanical lateral distal tibia angle, mMA: Mechanical Malleolar Angle, MHA: Malleolar Horizontal Orientation Angle, TPHA: Tibia Plafond Horizontal Orientation Angle, TTTA: Tibio Talar Tilt Angle

At the level of the ankle, we measured the following radiographic parameters (Fig. 4):

- Anatomic Lateral Distal Tibia Angle (aLDTA)
- Mechanical Lateral Distal Tibia Angle (mLDTA)
- Mechanical Malleolar Angle (mMA)
- Malleolar Horizontal Orientation Angle (MHA)
- Tibia Plafond Horizontal Orientation Angle (TPHA)
- Tibio Talar Tilt Angle (TTTA)

### Statistical analysis

Continuous variables were presented as mean ± standard deviation or range. Comparison between means was performed using the Wilcoxon test. A *p* value of < 0.05 was considered statistically significant. SPSS version 24 (IBM, Armonk, NY, USA) was used. A posthoc analysis was performed to ensure sufficient power to address the primary research question. Given the sample size of 27 patients, an effect size of 1.7 and an alpha error of 0.05, the power of the study was calculated to be 95%.

## Results

### Cohort demographics

The examined cohort of patients included 27 legs of 26 patients undergoing supracondylar external rotation osteotomy of the femur due to torsional malalignment of the femur and corresponding symptoms. The mean age was 32 (19–40) years. There were 21 female and 5 male patients. Radiologic imaging for alignment control measurements was obtained six to twelve months postoperatively. At this time, all osteotomies showed bony consolidation.

### The effect of supracondylar external rotation osteotomy on bony alignment of the leg

Surgery lead to significant changes regarding femoral torsion and coronal limb alignment, which was measured using the HKA, mLDFA, and TPHA along with several angles around the ankle (Table 1). Other measures did not change significantly: AMA (6.60 ± 0.87 preop, 6.79 ± 0.45 postop, mLPFA (90.32 ± 2.49 preop, 90.23 ± 2.36 postop), mMPTA (87.31 ± 2.01 preop, 87.43 ± 2.60 postop).

**Table 1:** Radiographic measures in patients undergoing supracondylar external rotation osteotomy of the femur

Radiographic measure	Preoperative	Postoperative	∆	*P*-value
Femoral torsion [°]	31.27 ± 4.00	17.43 ± 5.06	13.84°	< 0.001
Hip-Knee-Ankle angle [°]	2.40 ± 1.06	0.00 ± 1.26	-2.40°	< 0.001
mLDFA [°]	89.30 ± 1.80	86.97 ± 2.21	-2.33°	< 0.001
mMPTA [°]	87.31 ± 2.01	87.43 ± 2.60	0.12°	n. s
mLDTA [°]	90.36 ± 1.58	89.88 ± 1.09	-0.48°	n. s.
aLDTA [°]	89.24 ± 1.31	88.90 ± 1.01	-1.76°	n. s
mMA [°]	96.40 ± 2.10	90.76 ± 2.23	-5.64°	< 0.001
MHA [°]	14.00 ± 2.00	11.10 ± 2.01	-2.90°	< 0.001
TPHA [°]	1.30 ± 2.02	-1.30 ± 2.28	-2.60°	< 0.001
TTTA [°]	0.0 ± 0.0	0.0 ± 0.0	0.0°	n. s

*mLDFA* Mechanical lateral distal femoral angle*, mMPTA *Mechanical medial proximal tibial angle*. mLDTA *Mechanical lateral distal tibia angle*, aLDTA *Anatomic Lateral Distal Tibia Angle*, mMA *Mechanical Malleolar Angle*, MHA *Malleolar Horizontal Orientation Angle*, TPHA *Tibia Plafond Horizontal Orientation Angle*, TTTA *Tibio Talar Tilt Angle

**Fig. 5:**
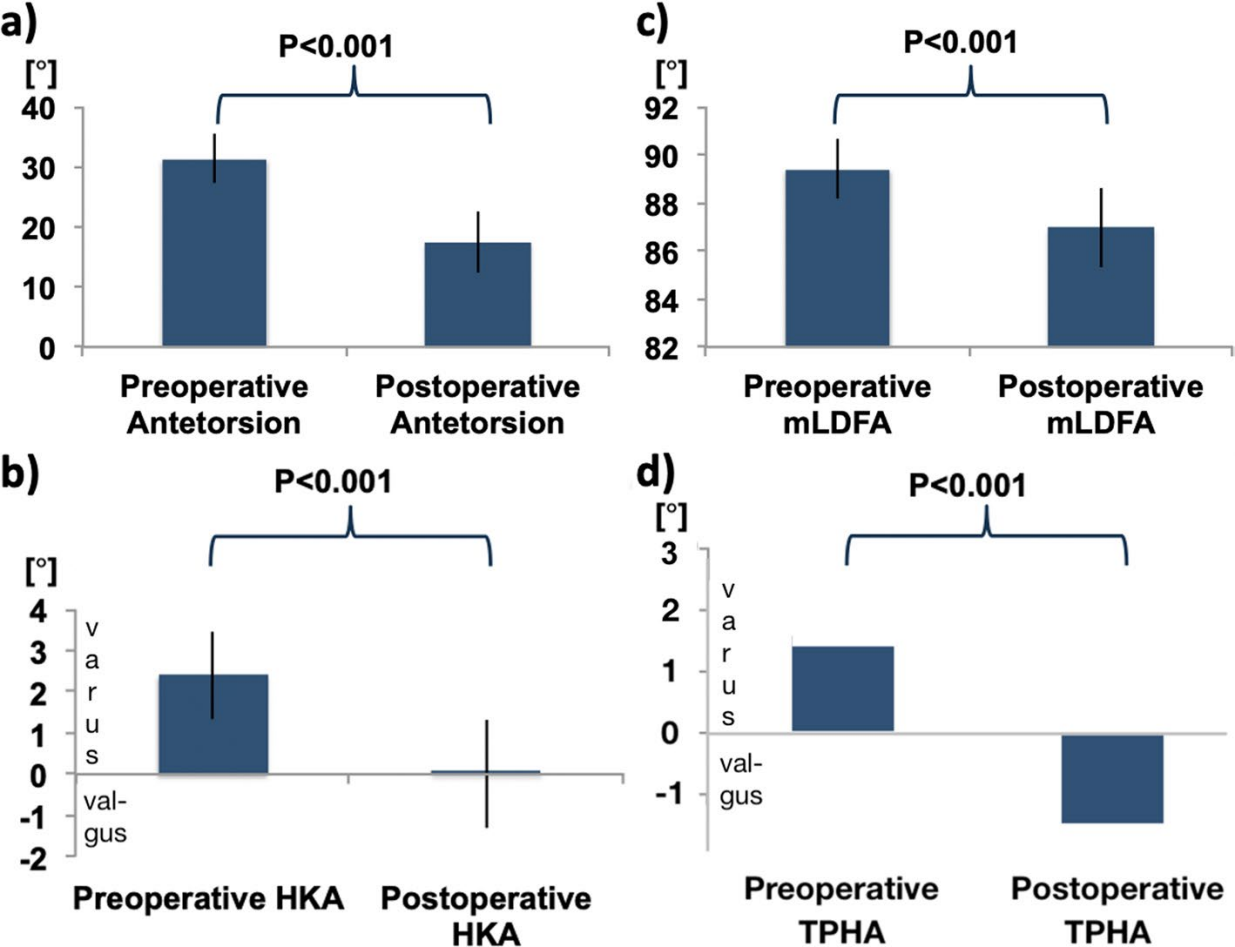
Supracondylar external rotation osteotomy of the femur: radiographic measures pre- and postoperatively. **a** Femoral antetorsion. **b** HKA. **c** mLDFA. **d** TPHA. HKA: Hip Knee Ankle angle, mLDFA: Mechanical lateral distal femoral angle, TPHA: Tibia plafond horizontal orientation angle

Supracondylar external rotation osteotomy led to the following measured changes illustrated in Fig. 5 a-d: reduction of femoral antetorsion (*p* < 0.001) and valgisation of the long-leg axis (*p* < 0.001) as demonstrated by the HKA angle, the mLDFA, and the TPHA (Table 1).

## Discussion

The most important finding of this study shows that a supracondylar external rotation osteotomy leads to valgisation of the coronal ankle alignment. This was demonstrated by the change in the TPHA on the long-leg standing X-ray (Fig. 5, Table 1). This is likely due to the resulting valgus alignment of the femur in the frontal plane.

This study was based on the idea that a change in orientation of the femoral antecurvature could alter the axis of the limb. Given that a long-leg standing X-ray is performed with the knees pointing forward, it can be deduced that the X-ray represents a natural illustration of a standing position of the lower extremity. Therefore, one must understand that the hip and femur rotate internally in relation to the femoral condyles and the convex side of the femoral antecurvature rotates from anterior to medial, resulting in a reduction of the mLDFA in an external rotation osteotomy of the distal femur. A bony valgus alignment of the femur is therefore created.

We further demonstrated that this effect of valgisation is also apparent at the ankle. This alters not only kinematics, but it must also influence the distribution of joint reaction forces.

In practice, supracondylar external rotational osteotomy was performed to reduce femoral antetorsion. On the operating table, external rotation of the distal femur fragment at first seemingly leads to a varisation of the leg on a two-plane X-ray, especially if the knee joint is slightly flexed. After osteotomy, as the patient walks with the foot directed straight forward, the patient rotates the hip joint inwards. As the osteotomy was performed distal to the antecurvature of the femur, the whole antecurvature also is rotated inwards and, as a matter of fact, this leads to a valgisation of the long-leg axis.

Regarding the ankle joint, unintentional valgisation might deteriorate biomechanics especially in patients with ligamentous instability. In the study cohort, the TTTA was 0° in all patients pre- and postoperatively. This could be different and changed by valgisation in unstable ankle joints, leading to new or aggravated symptoms of instability and pain.

The clinical findings of the study may further be seen when considering the site of the osteotomy. If valgisation is to be prevented, it would be helpful to perform rotational correction of high femoral antetorsion proximal to the level of the femoral antecurvature. An intertrochanteric osteotomy could have different effects on the coronal alignment that still need to be investigated.

Meticulous preoperative planning of the rotational osteotomy paying attention to all important angles especially around the knee and ankle is important to avoid unintentional creation of unphysiological angles outside the normal range [10].

The findings of this study are supported by an experimental study published earlier using a computer model with simulated external rotation osteotomies at different locations along the femur. The authors found a valgisation effect in distal (supracondylar) osteotomies. This alteration of the long-leg axis in the frontal plane was pronounced in cases with exceptional high femoral antecurvature [5].

Today, in the era of subspecialization, it is common practice for the hip surgeon to correct maltorsion of the femur proximally [1, 3, 4, 8, 14–17]. The specialized knee surgeon might tend to correct the same deformity distally, at the supracondylar site [18–20]. But this might not be the best option for every individual case given the fact that patients presenting with patellofemoral pain commonly have a valgus deviation, which shouldn’t be aggravated by external rotation osteotomy at the distal end of the femur [21].

So, not only in symptomatic knees and hips, but also in patients with disease of the ankle, the adjacent joint should be examined and the long-leg axis should be analyzed as part of the routine preoperative workup.

The main limitation of the study could be seen in the fact that a 3-dimensional reality has been simplified using 2-dimensional radiography. This problem would require complex imaging in a functional standing position. Given that the aim of this study was to prove the concept in a feasible standard clinical setting, the authors agreed on the sufficiency of the design chosen in this study. However, because of possible confounders to the postoperative alignment evaluated with 2-D X-ray in a standard clinical-like setting, the results of this study should be confirmed in future studies using 3-D space analysis.

## Conclusions

Supracondylar external rotation osteotomy of the femur lateralizes the weight bearing line by valgisation. As the TPHA is decreased, the foot reacts by inversion and supination. This should be taken into account when indicating and planning an isolated external rotation osteotomy at the distal femur in patients with a preexisting valgus alignment of the lower leg or restricted mobility of the subtalar joint.

## Data Availability

The datasets generated and analyzed during the current study are available from the authors on reasonable request.

## Abbreviations

AMA: Anatomic Mechanical Angle of the femur
HKA: Hip Knee Ankle angle
mTFA: Mechanical tibio-femoral angle
mLDFA: Mechanical lateral distal femoral angle
mLPFA: Mechanical lateral proximal femoral angle
mMPTA: Mechanical medial proximal tibial angle
aLDTA: Anatomic Lateral Distal Tibia Angle
mLDTA: Mechanical Lateral Distal Tibia Angle
mMA: Mechanical Malleolar Angle
MHA: Malleolar Horizontal Orientation Angle
TPHA: Tibia Plafond Horizontal Orientation Angle
TTTA: Tibio Talar Tilt Angle
